# Digitalization in management accounting and control: an editorial

**DOI:** 10.1007/s00187-020-00300-5

**Published:** 2020-04-23

**Authors:** Klaus Möller, Utz Schäffer, Frank Verbeeten

**Affiliations:** 1 University of St. Gallen, St. Gallen, Switzerland; 2 WHU - Otto Beisheim School of Management, Vallendar, Germany; 3 University of Amsterdam, Amsterdam, The Netherlands

**Keywords:** Digitalization, Management accounting, Controller roles, Future of finance

## Abstract

Digitalization has the potential to disrupt the management accounting domain. It may not only affect the digital landscape of the organization and the associated business models, but also management accounting and control practices as well as the role of the controller. This editorial discusses these developments by introducing the concept of digitalization and describing its impact on the field of management accounting and control.

Digitalization is the use of digital technologies to change a business model and provide new revenue and value-producing opportunities—the process of moving to a digital business (Gartner, 2020). As such, it has affected all kinds of business activities, including business models and supply chains, as well as support functions such as human resources and accounting. Digitalization enables various new forms of cooperation between companies, suppliers, customers, and employees, leading to new product and service offerings. At the same time, digitalization is a challenge for incumbent companies, as it requires them to reflect on their current strategy and to explore new business opportunities. In the finance function, digitalization has resulted in the automation and robotization of routine processes, the introduction of business intelligence, and the application of data analytics. Digitalization is affecting our daily lives as well as the role of controllers.^1^

## Research in digitalization

Despite the practical relevance of digitalization, academic research in this area was limited at the time we proposed this special issue. We noticed a large gap between theory and practice: academic papers hardly discussed the effects of digitalization on the finance function, while in our meetings with practitioners this was generally the only topic on the agenda. Many practitioners indicated that the potential impact on management accounting practices and the finance function was huge, with several companies having special transformation departments (for example in marketing, human resources, and finance) to guide the digital transition.

Although quite a few papers have been published in this area since then and authors such as Moll and Yigitbasioglu (2019) as well as Rikhardsson and Yigitbasiglu (2018) have provided overviews of the literature, we still observe that digitalization is only about to enter the scholarly debate. Most of the papers are largely conceptual (e.g. Bhimani & Willcocks, 2014; Quattrone, 2016; Arnaboldi, Busco, & Cuganesan, 2017a, b; Appelbaum et al., 2017), with some case studies (Arnaboldi, Azzone, & Sidorova, 2017a, b) and empirical analyses (Labro et al., 2019; Oesterreich et al., 2019). The field still appears dominated by consultants and practice pioneers (e.g. McKinsey, 2018; Deloitte, 2020). We believe that the development is too important for academia to leave it at that. The topic deserves a careful definition (what exactly are we talking about?), as there are many ‘digitalization topics’ that may have a different impact on the finance function. In addition to this, an overview and structuration of the field as well as conceptual ideas and reflection may be required.

## Impact of digitalization on the finance function

Where and how does digitalization affect the finance function? This content has mainly been fueled by discussions with practitioners in the field, but also developed from conversations with fellow academics at conferences, seminars, and other encounters. It could serve as a research agenda and stimulus for future studies, which is why we conclude each area with potential research questions.

### Strategy implementation and control

As the ‘economic conscience’ of the organization, controllers should be aware of the (long-term) viability of an organization, and therefore its digital strategy. Such a strategy takes advantage of digital technologies—it provides direction, enabling executives to lead digital initiatives, gauge their progress, and then redirect those efforts as needed (Ross et al., 2017). Controllers should play an active role in addressing digital opportunities and corresponding changes in business models and organizational strategies. That has many implications. Controllers not only need to develop and adapt new KPIs, but also flexible steering approaches (e.g. the Objective and Key Results system) and new portfolio techniques, mixing traditional with digital business models. In addition, traditional capital budgeting or investment control approaches might be inadequate in the context of exponential growth fueled by digital products, platform strategies, and network economies.

Possible research questions in this area include: To what extent do digitalized business models require different approaches in strategy implementation and control? Which management control systems can be used or how should they be adapted in a digital context? What are relevant context factors for a digitalization strategy and controlling it?

### Financial planning and analysis

Some companies have already introduced data analytics and automated forecasting technologies, using (or combining) time series techniques, machine/deep learning, and/or simulation. Key challenges include identifying and adequately applying appropriate techniques and drivers, and—even more importantly—the right combination of ‘(wo)man and machine’ in the application process. Especially in the light of structural breaks (such as the coronavirus/Covid-19 crisis) it seems to become evident that a combination of human judgment and business acumen with the extensive use of data and technology are key. Complete automation will likely only be effective in niches with clearly defined and understood processes.

Possible research questions include: What will be the impact of specific digital techniques on particular finance function processes? How can drivers for planning, forecasting, and simulation be identified, used, categorized, analyzed, and optimized? How can the interplay between ‘(wo)man and machine’ be designed? Which behavioral biases can be mitigated or can arise with the use of digital technologies?

### Reporting

Relevant and reliable data from a trustworthy, secure database should be the foundation of every decision. Creating and maintaining such ‘a single source of truth’ is a core responsibility of controllers that is, however, increasingly challenged by data scientists and other functions like IT. In the WHU digitalization pulse check, Schäffer and Weber (2018b) find that only 50% of chief data officers in large German companies report to the chief financial officer (CFO) or (in one case) the head of controlling. In other words, in half of the companies, the person ultimately responsible for data quality does not report to the person who traditionally claims to be the company’s single source of truth regarding financial data and the interpretation thereof. In addition, new information routines might lead to a more decentralized, self-service-based reporting and decision-making environment that may change the nature of control as well as the role of controllers. The use of chat bots and other robotic process automation techniques can create efficiency gains, but requires sound governance.

The challenges are manifold and so are the opportunities for research: What are effective digital reporting designs, process, structures, and governance systems? What are preconditions for self-controlling and self-reporting solutions? Which behavioral issues occur in digitalized reporting? How does a self-service reporting system affect managerial decision-making, and how does it affect the relevance of the controller? How are companies dealing with data governance?

### Competencies, roles and organizational structure

Given the challenges mentioned above, the finance function as well as individual controllers may need to develop new competencies. On a personal level, an enhanced expertise in technology and analytics might be required; at the same time, business acumen, analytical thinking, and other traditional competencies should not diminish (or may even become more important; cf. Kolthof et al., 2017b; Schäffer & Brueckner 2019). On an organizational level, the finance function will likely face a reduction in size (in the number of full-time equivalents; see Frey & Osborne, 2017; Schäffer 2017). This should however not restrict the effectiveness and impact of the function. On the contrary, new opportunities as well as new roles emerge (see Schäffer & Brueckner 2019).

Possible research questions include: Which competencies are required in a more digitalized context? What are effective strategies for competence building, transfer, and governance? What is the impact of digitalization on the roles of controllers? Which contextual factors are enabling or hindering a digitalized finance function? How should a digitalized finance function be structured by means of processes, responsibilities, and IT resources?

### The diffusion process

In the last couple of years, scholars, consultants, and practitioners have postulated a fundamental transformation of the finance function due to digitalization and increasing globalization (cf. Schäffer, 2017; Schäffer & Weber, 2016; Bhimani & Willcocks, 2014). Not surprisingly, Schäffer and Weber (2016) found empirical evidence that CFOs and controllers in Germany increasingly expect the finance function in their company to change. They conducted three studies on the future of controlling (2012, 2015, and 2018a). In the first survey, the average difference between the perceived importance of the top ranked controlling trends in 2011 and the importance expected in five years from then was 0.39 points (on a 7-point Likert scale). The respective delta points increased to 0.73 in 2014 and 1.00 in the 2017 study; the latter increase being primarily driven by trends relating to digitalization.

Most finance functions in large companies are not as advanced in their digitalization efforts as the commonplace c-suite rhetoric and the high expectation of change might suggest. This is reflected in the third WHU study on the future of controlling as well as a further study Schäffer and Weber conducted, namely the first WHU digital pulse check (Schäffer & Weber, 2018b). The authors consistently found that actual implementation is still in its infancy: 50% of the controllers surveyed indicated that their company has no digitalization strategy for controlling, while 30% reported the presence of only a preliminary strategy and a mere 6% of companies reported a relatively mature digitalization strategy in controlling. In addition, only 12% of respondents consider their company’s financial investment in the digitalization of controlling to be sufficient. Similarly, in the Netherlands Kolthof et al. (2017a) found that robotic process automation (RPA) is used in transaction processing for less than 50% of the activities. In addition, only in a minority of observations business analytics is used for financial planning and analysis.

Clearly, changes in finance function practices fall short of what has been suggested in earlier years. For instance, the expectations that CFOs and controllers reported in 2012 regarding managers’ access to data were consistently not met in 2017. Similarly, respondents’ prognoses in 2014 regarding the prevalence of the business partner concept in 2019 also appear to be unrealistic given the values in 2017. According to Schäffer and Weber (2018a), the adaptation processes in both instances require more time than the controllers in their three studies on the future of controlling originally expected.

Other findings in the aforementioned study by Schäffer and Weber provide empirical evidence that digitalization in the finance function, despite all the lip service and high expectations, is still in its infancy:

- In 2017, assessments of the importance of the trends ‘business analytics,’ ‘digital business models,’ ‘self-service reporting,’ ‘agile management,’ and ‘digital literacy’ range only between 3.2 and 4.2 on a 7-point Likert scale.
- Two thirds of the respondents perceive deficits in terms of data quality and accessibility. With regard to IT system integration, this number is even higher at 80%.
- Business analytics is used intensively in only a fraction of controller functions—5% of respondents reported intensive use.

Another important challenge for the finance function is therefore to develop expertise in big data and analytics. However, most controllers are not inherently competent in this area. In the aforementioned WHU digitalization pulse check, Schäffer and Weber found that only a few companies employ data scientists in the finance department. In the companies they surveyed, data scientists typically worked in IT and operations and only 22% of companies that employ data scientists did so in the controlling function. The findings also indicate relatively little interaction between controllers and data scientists, regardless of whether the latter work in controlling or elsewhere. According to respondents, controllers and data scientists work closely together in only 17% of companies in the sample of the WHU digitalization pulse check (Schäffer & Weber, 2018b).

So why does it take so long for the postulated transformation of the controlling function to get underway? First of all, we know from numerous studies that processes of fundamental change take time—at least when the changes are to be sustainable and more substantial than merely scratching the surface (Frey & Osborne, 2017). For example, Pritsch (2000) showed that it took at least 25 years—an entire generation—for the NPV method to become widespread (cf. Pritsch, 2000). In addition, the call for a new and more proactive controller role is not new, but can be traced back to the 1970s (see Henzler, 1974; Zünd, 1985; Siegel, 1999). However, many controllers still seem reluctant to act as business partners and the concept is far from being intern internalized by the majority of controllers in the field. And finally, the study by Schäffer and Matlachowsky (2008) demonstrates that even a structured implementation project provides no guarantee that an instrument’s use (specifically the balanced scorecard) will be securely anchored long-term and that this process often spans many years. In all three examples, sustainable change in the finance function is therefore a long process that cannot be measured in months or a few years. Why should this be any different in the case of digitalization?

One potential reason is the way top management teams prioritize business units and corporate functions in the pursuit of digitalization. However, anecdotal as well as more substantive empirical evidence seems to show that CEOs tend to prioritize areas that directly add to corporate value creation, such as marketing and supply chain. Internal service providers tend to be deprioritized, resulting in the finance function being a relative latecomer as far as digital transformation is concerned. This may have implications for the overall status of the finance function and for its role in digital transformation. Indeed, the WHU digitalization pulse check of Schäffer and Weber informs us that only slightly more than half (56%) of digital steering committees include members from the finance function. A relatively large portion of German companies therefore appear to address digitalization topics without directly involving the finance function.

## Articles in this issue

Summing up, we believe that the digitalization of management accounting and control has substantial potential to transform our research field, and that the articles in this issue provide an interesting avenue to start the dialogue. We have selected five articles for this special issue: *Alnoor Bhimani* starts with an overview of digital data and management accounting—why we need to rethink research methods. The next two articles focus on the effects of digitalization on core controlling processes: budgeting and reporting. *Mareike Bergmann, Christian Brück, Thorsten Knauer and Anja Schwering* deal with the digitalization of the budgeting process. Based on an empirical study, they research the determinants of the use of business analytics and its effect on satisfaction with the budgeting process. Lisa Perkhofer, Conny Walchshofer and Peter Hofer focus on visualizing the design of big data and investigate the effect of visualization type and interaction in an empirical study. Then we enlarge the perspective on the overall control system in two articles with a more holistic approach: Thorsten Knauer, Nicole Nikiforow and Sebastian Wagener deal with information systems, but on a more abstract level, by analyzing determinants of information system quality and data quality. Finally, Gianluca Vitale, Sebastiano Cupertino and Angelo Riccaboni show the interplay between formal and informal control systems, influenced by big data based on a case study.

We thank all contributors, reviewers, and especially the managing editor of the *Journal of Management Control*, Prof. Dr. Thomas Günther, for supporting this special issue.

The guest-editors of the special issue:

Prof. Dr. Klaus Möller

Prof. Dr. Utz Schäffer

Prof. Dr. Frank Verbeeten

## Footnotes

1 We use the term ‘controller’ rather than the equivalent ‘management accountant’ in this editorial. Similarly, we use the term ‘finance function’ rather than ‘management accounting and control function’ or ‘accounting and finance function.’
